# The electronic structure and optical properties of Mn and B, C, N co-doped MoS_2_ monolayers

**DOI:** 10.1186/1556-276X-9-554

**Published:** 2014-10-06

**Authors:** Wei-bin Xu, Bao-jun Huang, Ping Li, Feng Li, Chang-wen Zhang, Pei-ji Wang

**Affiliations:** 1 School of Physics and Technology, University of Jinan, Nan Xin Zhuang west road No. 336, Jinan, Shandong 250022, People’s Republic of China

**Keywords:** MoS_2_ monolayer, Mn-B co-doped, Mn-C co-doped, Mn-N co-doped, Electronic structure, Optical properties

## Abstract

The electronic structure and optical properties of Mn and B, C, N co-doped molybdenum disulfide (MoS_2_) monolayers have been investigated through first-principles calculations. It is shown that the MoS_2_ monolayer reflects magnetism with a magnetic moment of 0.87 μB when co-doped with Mn-C. However, the systems co-doped with Mn-B and Mn-N atoms exhibit semiconducting behavior and their energy bandgaps are 1.03 and 0.81 eV, respectively. The bandgaps of the co-doped systems are smaller than those of the corresponding pristine forms, due to effective charge compensation between Mn and B (N) atoms. The optical properties of Mn-B (C, N) co-doped systems all reflect the redshift phenomenon. The absorption edge of the pure molybdenum disulfide monolayer is 0.8 eV, while the absorption edges of the Mn-B, Mn-C, and Mn-N co-doped systems become 0.45, 0.5, and 0 eV, respectively. As a potential material, MoS_2_ is widely used in many fields such as the production of optoelectronic devices, military devices, and civil devices.

## Background

Layered transition metal dichalcogenides (TMD) belong to a well-defined chemical and structural family characterized by strong covalent intralayer bonding and weak van der Waals interactions between adjacent layers [1,2]. Transition metal oxides and sulfides have always been an interesting subject in experimental and theoretical works [3-9] due to their important role in lithium-ion batteries (LIB) [10], flexible electronic devices [11], photoluminescence [12], valleytronics [13,14], and field-effect transistors. Molybdenum disulfide (MoS_2_) monolayer contains hexagonal planes of Mo atoms lying between two hexagonal planes of S atoms, forming a sheet of S-Mo-S. Each Mo atom bonds with six neighboring S atoms through covalent bonds.

Cheng et al. [15] have found that the formation energy of substitutional doping is formidably large in graphene, rendering doping in this 2D material a challenging issue. It has been proved that a very thin MoS_2_ owns a good property of lubrication. It is mainly because the binding energy between S atoms and metal materials is so strong that MoS_2_ has a great adsorbability on the metal surface. MoS_2_ can also be used as a kind of desulfurization catalyst [16,17] for crude oil in the industry, indeed preventing the phenomenon of sulfur poisoning. Due to its good chemical stability, thermal stability, specific surface area, and high surface activity, MoS_2_ can be a utility material. Though its optical and electronic properties [18-24] have been discussed, MoS_2_ still has limitations in improving the optical property for the production of photodetectors in the industry. Doping in MoS_2_[25-27], as a typical 2D material, attracts more attention. Through a series of calculations, Mn doping and B (C, N) doping can improve the characters of MoS_2_[24,25]. In order to get more ideal characters of MoS_2_, we calculated three structures including Mn-B, Mn-C, and Mn-N co-doped MoS_2_ monolayers in this paper. The MoS_2_ monolayer co-doped with Mn-C reflects magnetism. However, the systems co-doped with Mn and B (N) atoms exhibit semiconducting behavior with bandgaps smaller than those of the corresponding primitive state. Mn-B (C, N) co-doping all make the optical absorption edges generate the redshift phenomenon for the MoS_2_ monolayer, which results in the enhancement of absorption for infrared light in the MoS_2_ monolayer. The redshift degree of the Mn-N co-doped system is the largest. This result may open a new route to MoS_2_ in optical device applications.

## Methods

**Figure 1:**
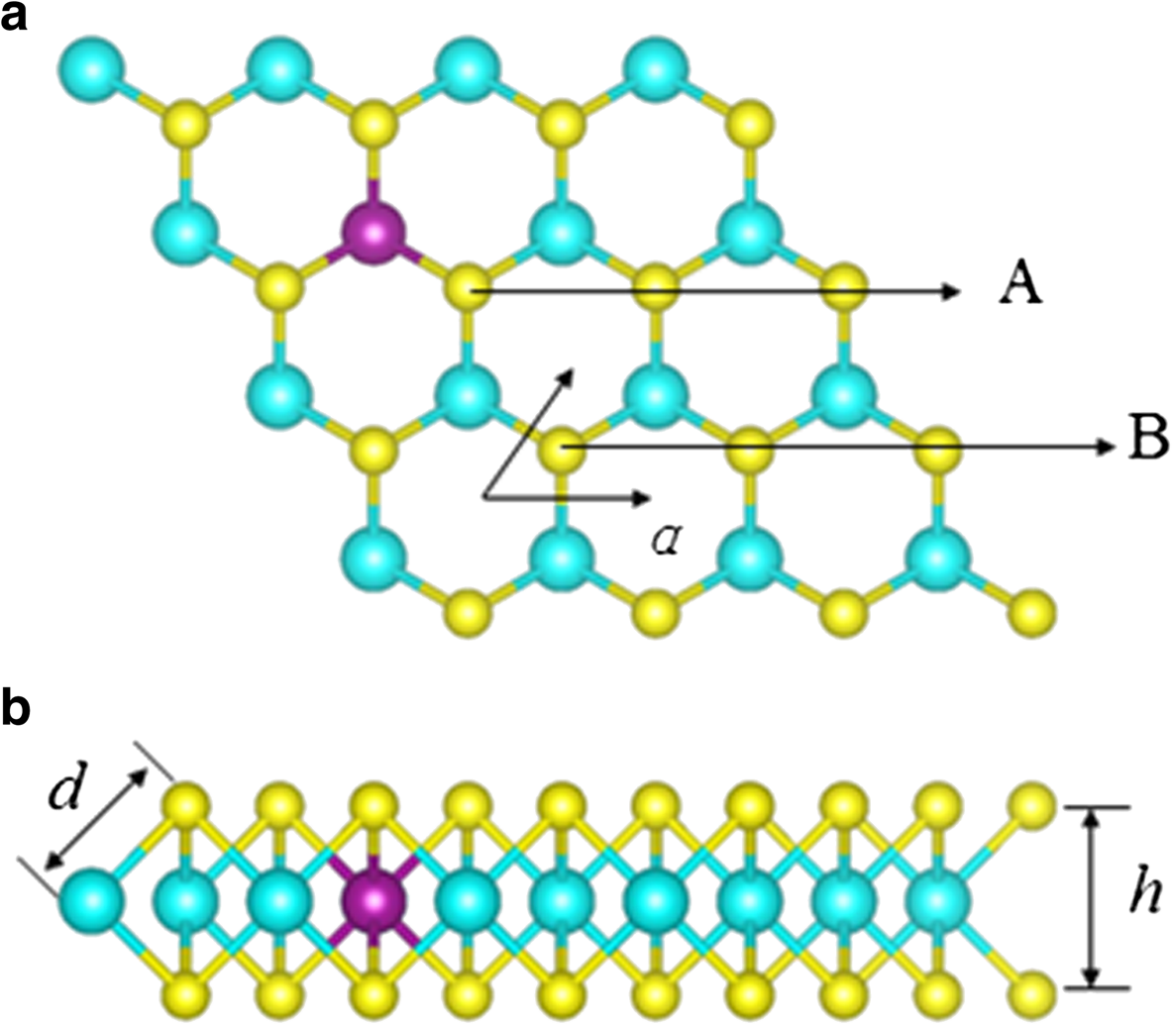
**Optimized geometric structures of the MoS**_**2 **_**monolayer from the top view (a) and side view (b).** The blue, yellow, and purple balls are Mo, S, and Mn atoms, respectively. B or C atom substitutes for the S atom at site A, and N atom replaces the S atom located at site B.

In this paper, we will discuss three co-doped structures: Mn-B, Mn-C, and Mn-N co-doped MoS_2_ monolayers, as shown in Figure 1. All of the computations are performed using the spin-polarized density functional theory with an all-electron linearized augmented plane wave method, as implemented in the WIEN2K simulation package [28], in order to investigate the electronic and optical properties of the MoS_2_ monolayer. The cutoff energy is 300 eV, and the muffin tin radius of Mo, S, Mn, B, C, and N is 1.45, 1.04, 1.40, 0.85, 0.70, and 0.65 Å, respectively. A generalized gradient approximation [29] is used to treat the exchange correlation potential, and relativistic effects are taken into account. In order to get comprehensive results about the Mn-3d orbit, the GGA + U method is also used. The 4 × 4 × 1 supercell of MoS_2_ with *a* = *b* =12.66 Å [30,31] is adopted through all the calculations, and the 2D MoS_2_ are located in the *x*-*y* plane with periodic boundary conditions and are modeled in a supercell with a vacuum space of at least 20 Å in the *z*-axis in order to avoid interactions between adjacent sheets. The Brillouin zone (BZ) is represented by a set of 6 × 6 × 1 *k*-points [32] for geometry optimization and for static total energy calculations. Structural relaxation is done until the forces on each atom are smaller than 10^-2^ eV/Å.

## Results and discussion

### Formation energy and crystal structure

The relative stability of the doped structure is determined from the formation energy and relates to the realization in experiments. Through co-doping, the formation energy can be calculated by the following general equation, which is inferred from some experiences of other semiconductor materials in any form [33-36]:

$$E_{\mathrm{form}}=\;E_{\mathrm{tot}}\left[X\right]-E_{\mathrm{tot}}\left[{MoS}_{2}\right]-\sum n_{i}E_{i}$$

*E*_tot_[MoS_2_] and *E*_tot_[*X*] represent the total energy of the primitive MoS_2_ monolayer and the total energy doped with impurities, respectively. *n*_i_ >0 means the number of atoms which are doped into the system, while *n*_i_ <0 means the number of atoms which are replaced from the MoS_2_ monolayer. *E*_i_ represents the energy of the single atom. The smaller the value of the formation energy, the greater the stability of the structure. The formation energy under the circumstances of Mn and B, C, N co-doped MoS_2_ monolayers is 7.42, 7.03, and 7.56 eV, respectively. Obviously, the case of the Mn-C co-doped system obtains the most stable state.

Due to the sandwich structure of MoS_2_, we put our point to the buckled height between two S atom planes (*h*), the length of Mo-S (*d*), and the S-Mo-S bond angle (*θ*) which are 3.16 Å, 2.42 Å, and 81.65°, respectively. As is known, the order of radius for nonmetallic atoms is N > C > B. Table 1 shows us that, with the increase of the radius, *θ* (S-Mo-S) becomes smaller and also the bond length between Mn and S becomes longer and *d* (Mo-*X*) becomes shorter. The buckled height *h* (S-S) under the circumstance of co-doping is smaller than that of the primitive state.

**Table 1:** **The crystal structure of the co**-**doped MoS**_**2 **_**monolayers**

	***h *** **(S-S)**	***θ *** **(S-Mo-S)**	***d *** **(Mn-S)**	***d *** **(Mn- *X *)**	***d *** **(Mo- *X *)**
Mn-B	3.15	81.88	2.29	2.01	2.12
Mn-C	3.14	81.44	2.32	1.93	2.05
Mn-N	3.14	81.34	2.33	4.95	1.99

The symbols *h*, *d*, and *θ* are the buckled height, bond length, and bond angle, respectively (*X* = B, C, N).

### Density of states

**Figure 2:**
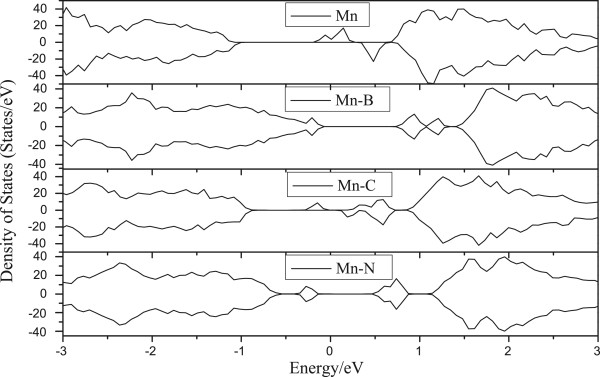
**Total density of states of Mo**_**15**_**MnBS**_**31**_, **Mo**_**15**_**MnCS**_**31**_**, and Mo**_**15**_**MnNS**_**31**_**.**

In Figure 2, we further present the total density of states (DOS) of all structures. Although the pure MoS_2_ monolayer is a nonmagnetic semiconductor, the Mn doping results in magnetic states with spin-up and spin-down branches being unequally occupied. The result well agrees with ref. [26]. From DOS, Mo_15_MnCS_31_ becomes a magnetic semiconductor, which spins up and spins down asymmetrically. Mostly in view of the orbital coupling between C-2 s, C-2p and Mn-3d, Mn-4 s, Mo-5 s, a series of local energy levels appear around the Fermi level.

However, both Mo_15_MnBS_31_ and Mo_15_MnNS_31_ are still semiconductors, which spin up and spin down symmetrically. In Mo_15_MnCS_31_, the role of Mn upon the conduction band is stronger than that in Mo_15_MnS_32_. This phenomenon is due to the Mn and C atoms sharing pairs of electrons. Meanwhile, the interaction between electric charges is reinforced and the polarization phenomenon generated. Consequently, the role of Mn-3d upon the conduction band around the Fermi level is reinforced.

**Figure 3:**
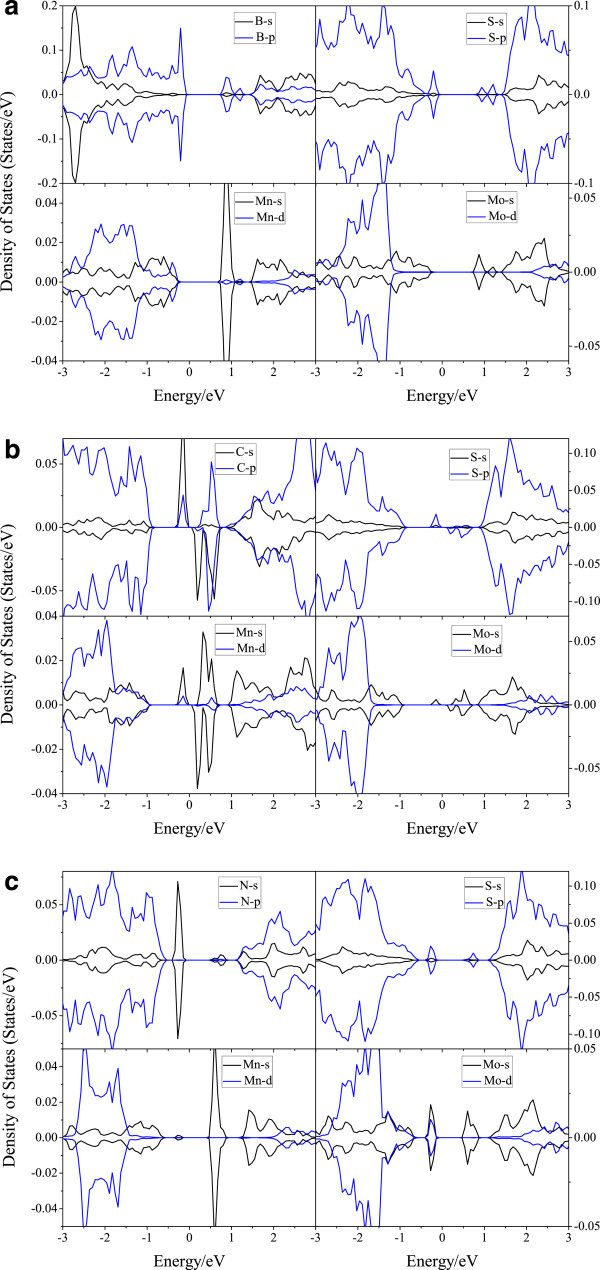
**P-DOS of Mo**_**15**_**MnBS**_**31 **_**(a), Mo**_**15**_**MnCS**_**31 **_**(b), and Mo**_**15**_**MnNS**_**31 **_**(c).**

To better understand the effect between different orbits, we demonstrate the partial density of states as shown in Figure 3. In Mo_15_MnBS_31_, the top of the valence band is contributed by B-2p and the bottom of the conduction band is determined by Mn-4 s and B-2p in the majority. Between -3 and -2.5 eV, the valence band is mainly determined by B-2 s and S-3p. The role of B-2 s upon the conduction band between 1.5 and 3 eV is more important than that of B-2p. When the MoS_2_ monolayer is co-doped with Mn-C, several local energy levels appear around the Fermi level. This phenomenon is mostly due to the orbital coupling between C-2 s, C-2p and Mn-3d, Mn-4 s. In Mo_15_MnNS_31_, the top of the valence band as well as the bottom of the conduction band is mainly contributed by N-2 s and Mn-4 s, respectively. N-2 s plays an important role between -0.4 and -0.1 eV in the valence band.

In order to realize the effect of the Mn-3d orbit deeply, the GGA + U method is used. The results show us that the orbital coupling between C-2 s, C-2p and Mn-3d becomes stronger as *U* increases. And the electronic transition between B-2p, C-2 s, C-2p, N-2 s and Mn-3d becomes more active. Above all, the role of Mn-3d enhanced. Considering the Coulomb repulsion between the electrons, the results become convincing.

### Energy bandgap

**Figure 4:**
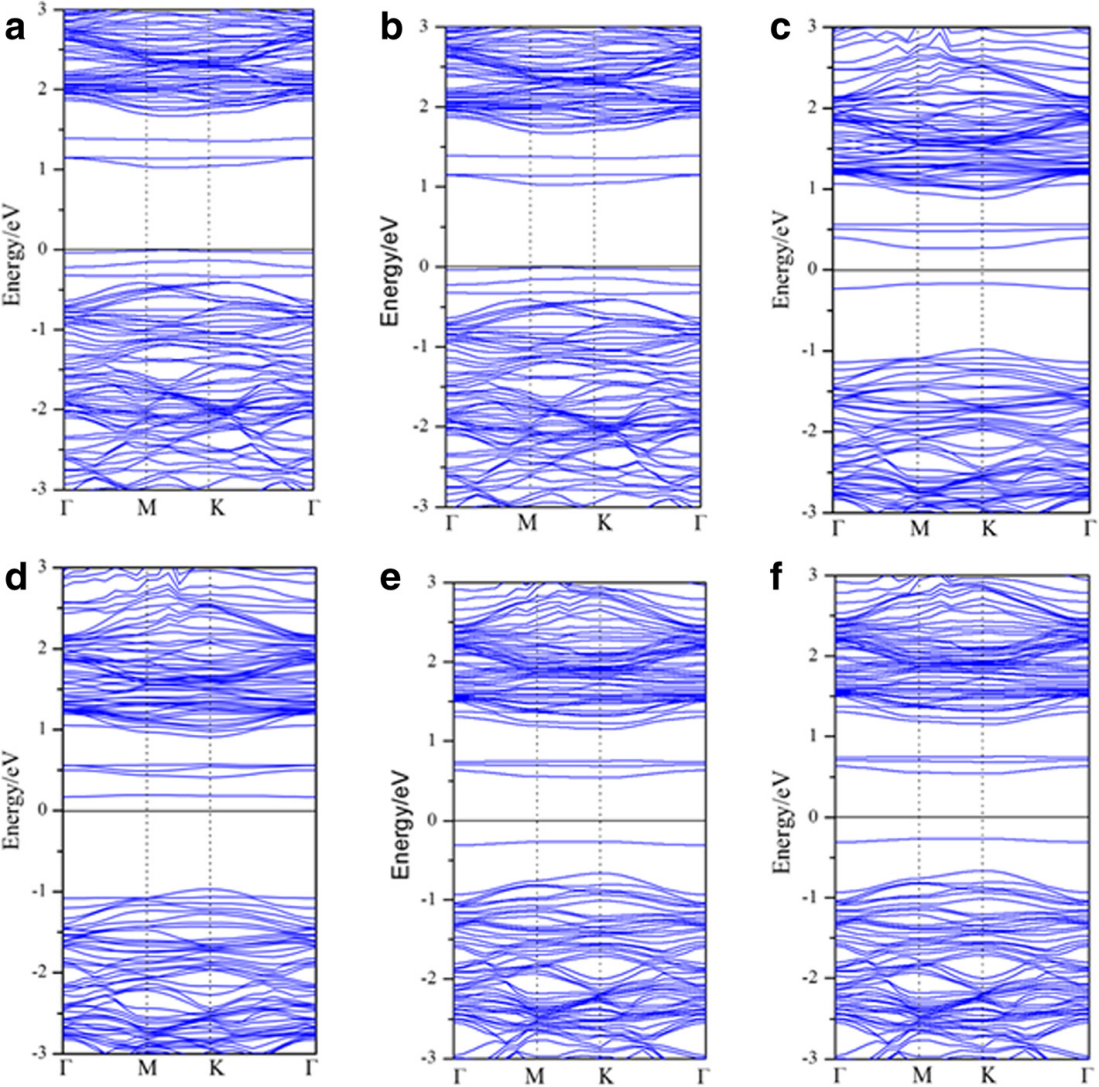
**The energy band of the doped structures. (a, b)** Mn-B co-doped MoS_2_ monolayer, **(c, d)** Mn-C co-doped MoS_2_ monolayer, **(e, f)** Mn-N co-doped MoS_2_ monolayer. The former of the pattern in pairs shows the energy band of spin-up, and the latter shows the band structure of spin-down.

The primitive MoS_2_ monolayer has a direct bandgap of 1.85 eV which is consistent with ref. [3]. The band structures are shown in Figure 4; Mn-B and Mn-N co-doping make the energy bandgap become smaller than before, where the bandgaps are 1.03 and 0.81 eV, respectively. And the Mo_15_MnNS_31_ transforms into an indirect semiconductor. Unsurprisingly, the Mn-B (N) co-doping cannot transform the spin state of the material, which makes the system stay in a nonmagnetic state. It is due to the effective charge compensation between Mn or B (N) atoms. But the Mn-C co-doped system reflects spin polarization and the magnetic moment is 0.87 μB. The generation of the magnetic moment is mainly because Mn provides one more electron than the Mo atom; when C substitutes for the S atom, it needs more electrons to make the 2p orbit saturated. In Figure 4c,d, a series of impurity bands appear around the Fermi level which results in the reinforcement of the light absorption and expands the absorbed region. These strong local lines come from the orbital hybridization between C-2 s, C-2p and Mn-3d, Mn-4 s, Mo-5 s, which provide more electronic states in energy space per unit. For Figure 4a,b, we can clearly see that the Fermi level shifts down, which means that Mo_15_MnBS_31_ can provide amounts of vacancies. Figure 4e,f indicates that the Fermi level shifts up and Mo_15_MnNS_31_ provides plenty of electrons. All of the co-doped systems gain different abilities of electronic transition between the top of the valence band (TVB) and the bottom of the conduction band (BCB). By comparing the energy band structures, the Mn-C co-doped MoS_2_ monolayer has a higher spin polarization. Orbital hybridization makes the valence exclusion effect stronger in Mo_15_MnCS_31_. The narrowness of the bandgap indicates that the absorbed region of this semiconductor becomes larger. This result is well consistent with the density of state before.

### Optical property

The optical properties of Mo_15_MnBS_31_, Mo_15_MnCS_31_, and Mo_15_MnNS_31_ all reflect the redshift phenomenon which leads to the MoS_2_ monolayer absorbing more infrared light. The optical property of the primitive state well agrees with studies before [37,38].

**Figure 5:**
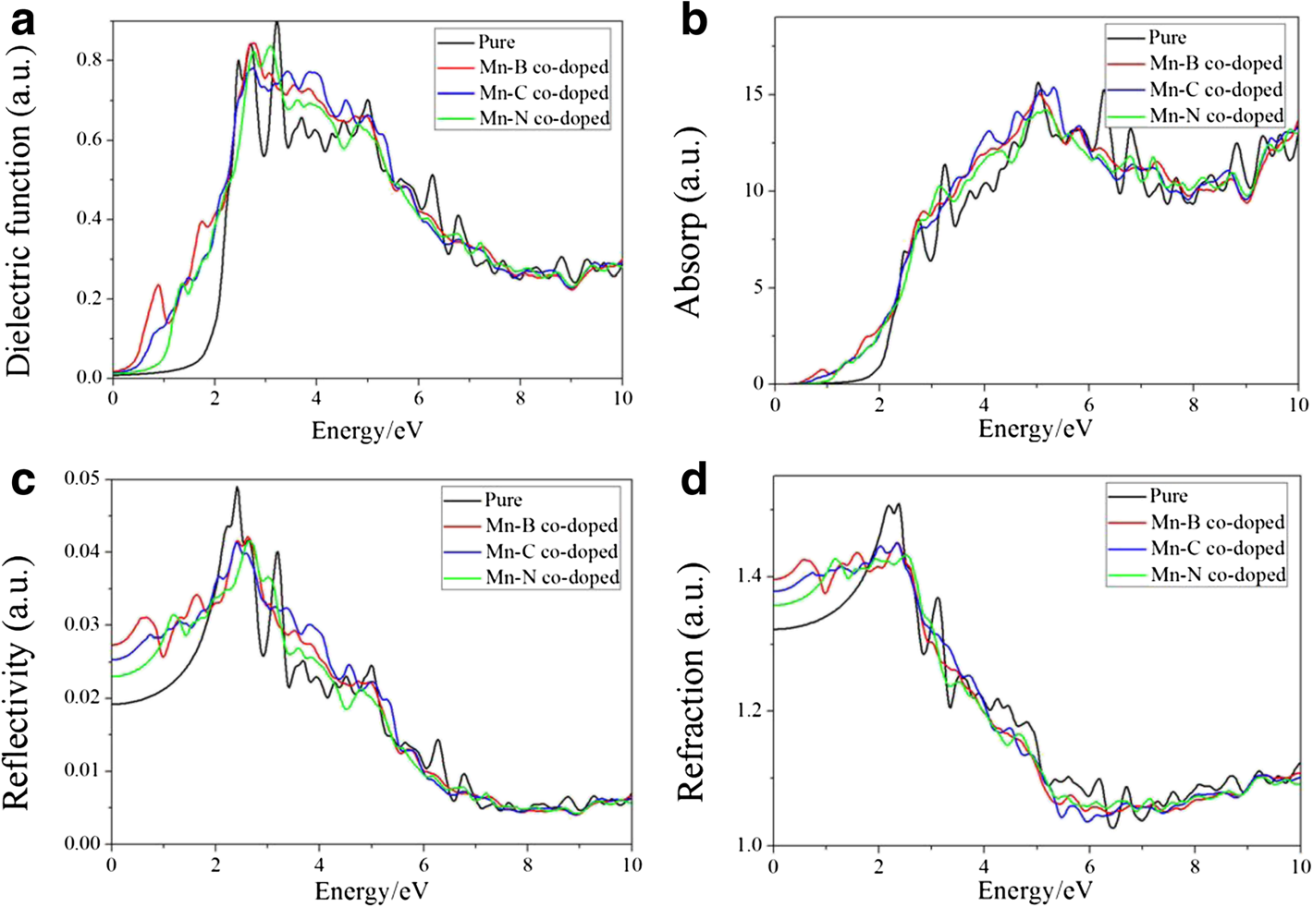
**The optical properties of Mo**_**16**_**S**_**32 **_**(black line), Mo**_**15**_**MnBS**_**31 **_**(red line), Mo**_**15**_**MnCS**_**31 **_**(blue line), and Mo**_**15**_**MnNS**_**31 **_**(green line). (a)** Dielectric function, **(b)** absorption, **(c)** reflectivity, and **(d)** refractivity.

In Figure 5a, Mo_15_MnBS_31_, Mo_15_MnCS_31_, and Mo_15_MnNS_31_ all emerge as a series of peaks between 0 and 2 eV because of the electronic transition between B-2p and Mn-4 s; C-2 s, C-2p and Mn-3d, Mn-4 s; and N-2 s and Mn-4 s, Mo-5 s near the Fermi level, respectively. In Mo_15_MnBS_31_, Mo_15_MnCS_31_, and Mo_15_MnNS_31_, the peaks of the dielectric function all reflect the redshift phenomenon in which the value of the peaks in the high-energy region becomes gentle and small and the whole tendency gains no more changes. However, the peak of the dielectric function for Mo_15_MnBS_31_ is the largest in the low-energy area. It illustrates that the electronic transition between B-2p and Mn-4 s is active during this region.

In Figure 5b, the absorption edge of the pure molybdenum disulfide monolayer is 0.8 eV, corresponding to the electrons which transfer from the conduction band to the valence band partially, which is in very good agreement with the experimental value [7]. The co-doped structures all reflect the redshift phenomenon, and the absorption edges of the Mn-B, Mn-C, and Mn-N systems become 0.45, 0.5, and 0 eV, respectively. And the value of absorption peaks decreased simultaneously. The redshift phenomenon shows us that the co-doped systems have better optical gas sensing property. Although the number of absorption peaks decreased, the energy range increased, which indicates that the wavelength range for the absorption became wider. In the high-energy region, the absorption of Mo_15_MnBS_31_, Mo_15_MnCS_31_, and Mo_15_MnNS_31_ is so little such that the MoS_2_ monolayer has high transmittance in the visible light region under these circumstances. These findings indicate that the pure MoS_2_ is more suitable to make a near-ultraviolet (6.0 ~ 6.5, 6.8 ~ 7.0, and 8.5 ~ 9.5 eV) photodetector than the MoS_2_ monolayer co-doped with Mo-B (C, N). But Mo_15_MnCS_31_ is the most suitable to make a near-ultraviolet (3.3 ~ 5.8 eV) photodetector. The Mn-B co-doped MoS_2_ monolayer is more suitable to make an infrared photodetector.

Figure 5c,d shows us the reflectivity and refractivity of the MoS_2_ monolayer. Mo_15_MnBS_31_, Mo_15_MnCS_31_, and Mo_15_MnNS_31_ all produce a series of peaks in the low-energy area and reflect the redshift phenomenon. These peaks are mainly due to the electronic transition of B-2p and Mn-4 s; C-2 s and Mn-3d, Mn-4 s; and N-2 s and Mn-4 s, respectively. Due to the active electronic transition between B-2p and Mn-4 s as well as the symmetry breaking induced by the Mn-B dopant, Mo_15_MnBS_31_ owns a maximum peak. On the contrary, in the infrared light region, Mo_16_S_32_ gets the minimum reflectivity. Hence, Mo_16_S_32_ obtains high transmittance in the infrared light region. The variation tendency of the primitive MoS_2_ monolayer in reflectivity is consistent with what Newaz has done [9].

## Conclusions

According to our calculation, the electronic structure and optical properties of Mn and B, C, N co-doped MoS_2_ monolayers have been investigated through first-principles. As is shown, the MoS_2_ monolayer co-doped with Mn-C reflects magnetism and the magnetic moment is 0.87 μB. It is due to the Mn providing one more electron than the Mo atom; when C substitutes for the S atom, it needs more electrons to make the 2p orbit saturated. However, the co-doped systems with Mn and B (N) atoms exhibit semiconducting behavior with bandgaps smaller than those of the corresponding pristine state because of the effective charge compensation between Mn and B (N) atoms. And the energy bandgaps are 1.03 and 0.81 eV, respectively. Mn-B (C, N) co-doping all make the optical absorption edges generate the redshift phenomenon for the MoS_2_ monolayer, which results in the enhancement of the MoS_2_ monolayer absorbing infrared light. The absorption edge of the pure molybdenum disulfide monolayer is 0.8 eV, where the absorption edges of Mn-B, Mn-C, and Mn-N co-doped systems become 0.45, 0.5, and 0 eV, respectively. Mo_15_MnCS_31_ is easier to achieve in the experiments than other structures. As a potential material, it is necessary to realize the tunable bandgap in the MoS_2_ monolayer by surface adsorption. Furthermore, our research will progress towards quantum transport simulation and tunneling transistors like silicene [39,40].

## Competing interests

The authors declare that they have no competing interests.

## Authors’ contributions

P-JW and C-WZ conceived the idea and designed the calculated model. W-BX carried out the electronic structure calculations and data analysis. PL, FL, and B-JH performed the analysis method of optical properties. All authors read and approved the final manuscript.
